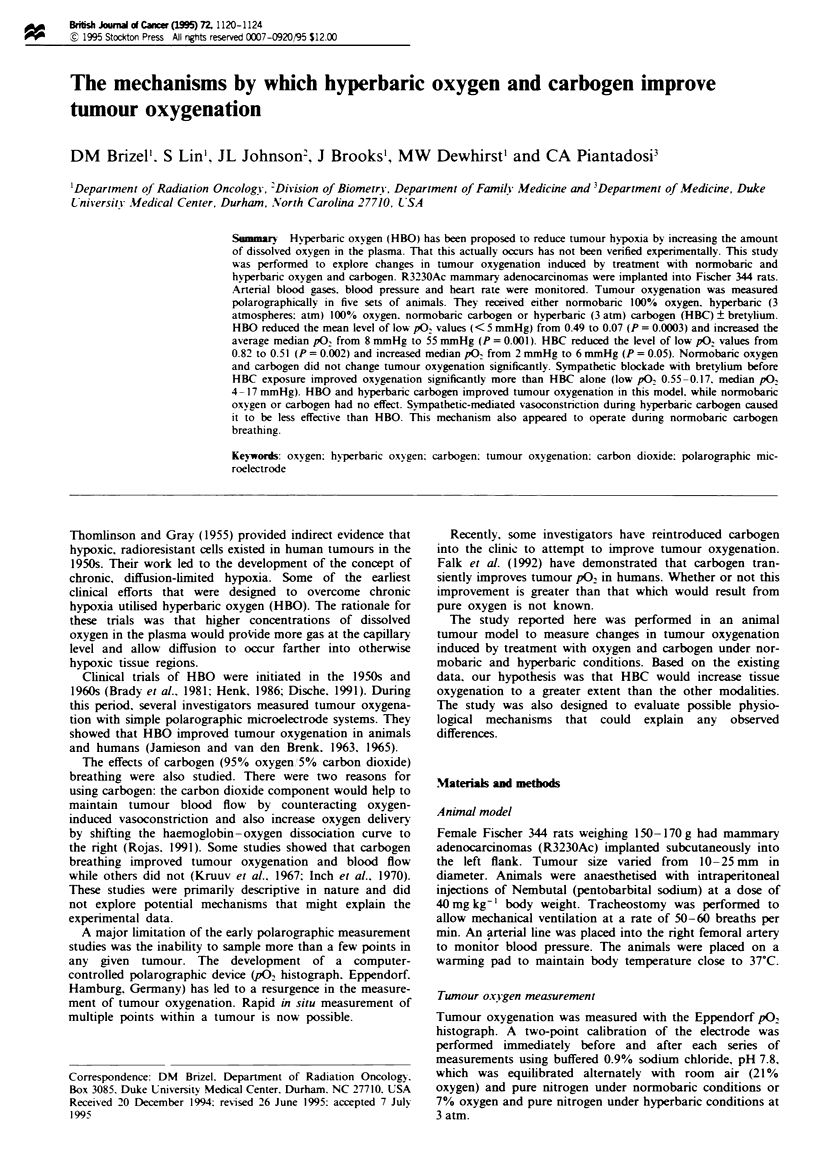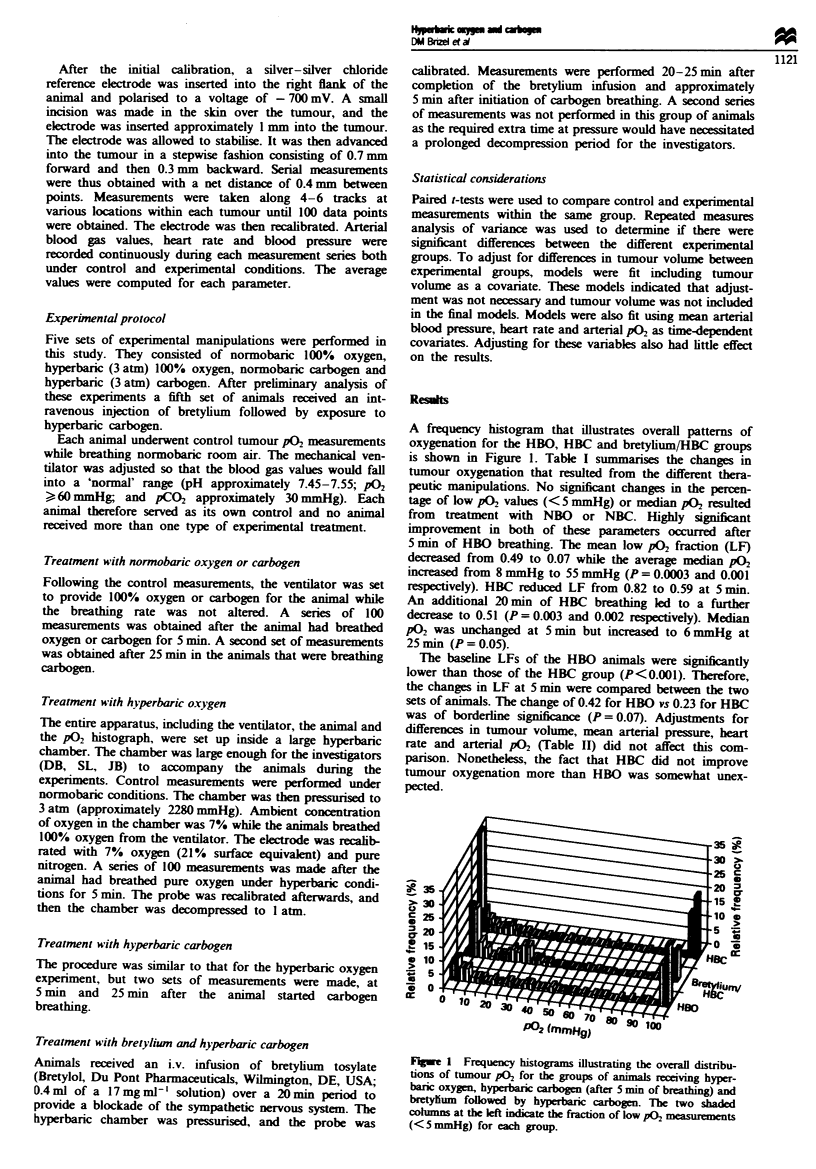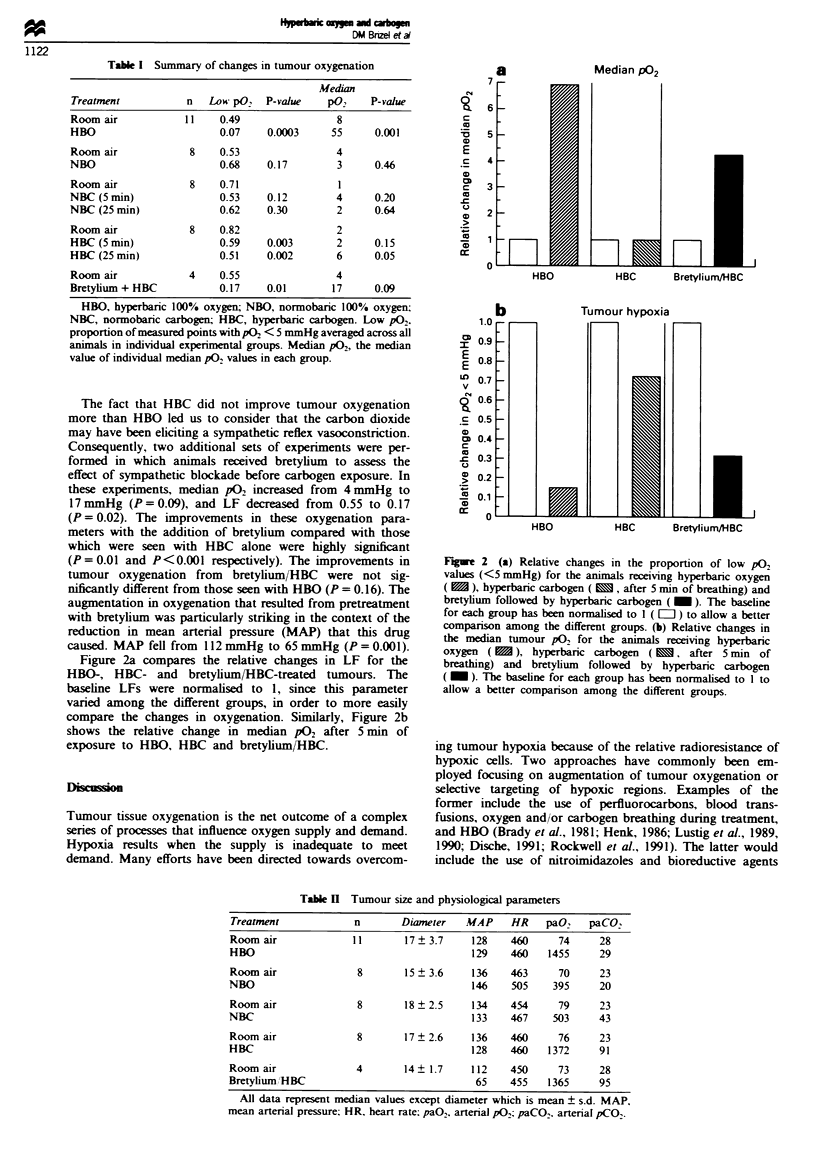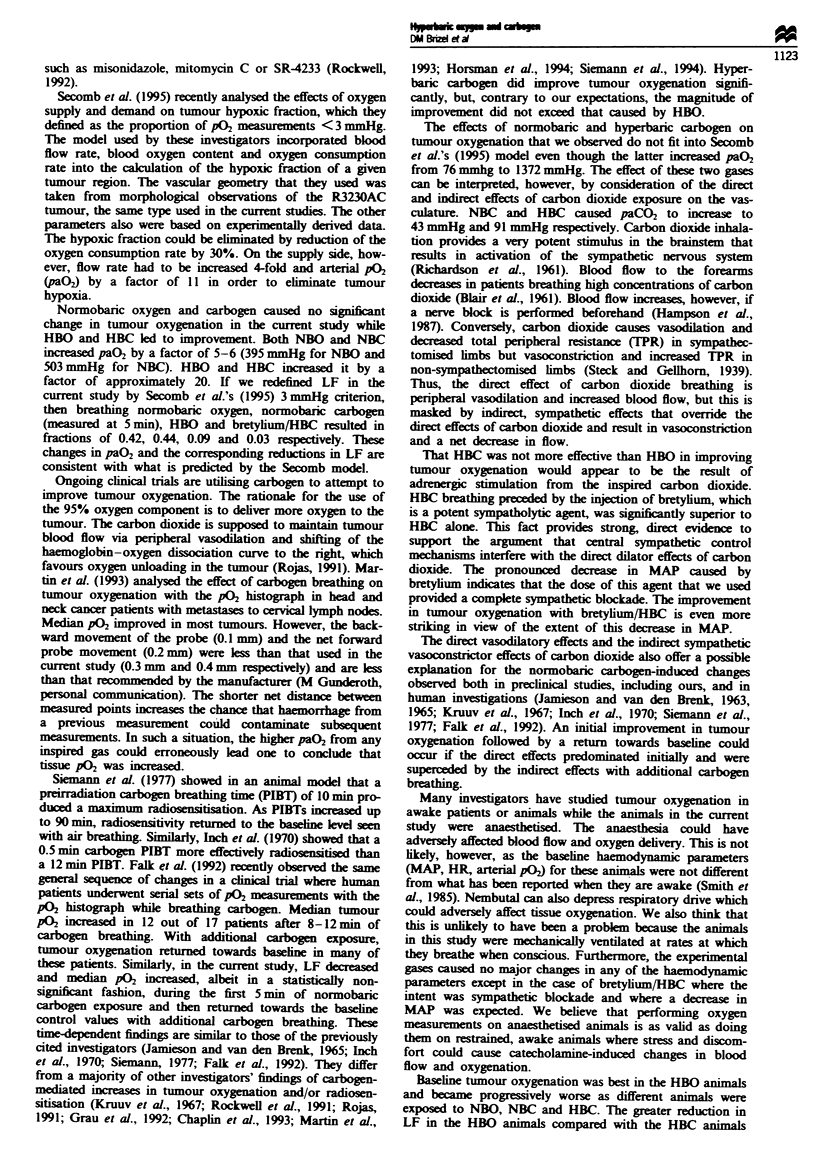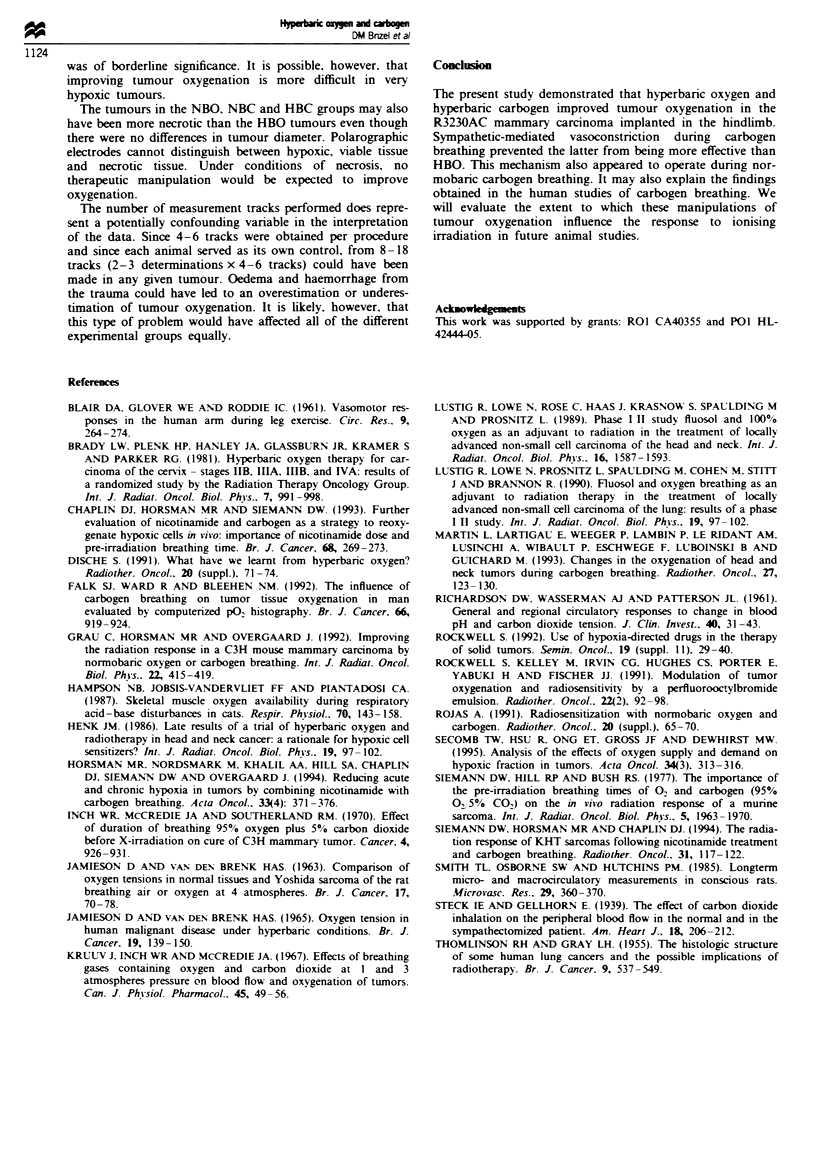# The mechanisms by which hyperbaric oxygen and carbogen improve tumour oxygenation.

**DOI:** 10.1038/bjc.1995.474

**Published:** 1995-11

**Authors:** D. M. Brizel, S. Lin, J. L. Johnson, J. Brooks, M. W. Dewhirst, C. A. Piantadosi

**Affiliations:** Department of Radiation Oncology, Duke University Medical Center, Durham, North Carolina 27710, USA.

## Abstract

Hyperbaric oxygen (HBO) has been proposed to reduce tumour hypoxia by increasing the amount of dissolved oxygen in the plasma. That this actually occurs has not been verified experimentally. This study was performed to explore changes in tumour oxygenation induced by treatment with normobaric and hyperbaric oxygen and carbogen. R3230Ac mammary adenocarcinomas were implanted into Fisher 344 rats. Arterial blood gases, blood pressure and heart rate were monitored. Tumour oxygenation was measured polarographically in five sets of animals. They received either normobaric 100% oxygen, hyperbaric (3 atmospheres; atm) 100% oxygen, normobaric carbogen or hyperbaric (3 atm) carbogen (HBC) +/- bretylium. HBO reduced the mean level of low pO2 values (< 5 mmHg) from 0.49 to 0.07 (P = 0.0003) and increased the average median pO2 from 8 mmHg to 55 mmHg (P = 0.001). HBC reduced the level of low pO2 values from 0.82 to 0.51 (P = 0.002) an increased median pO2 from 2 mmHg to 6 mmHg (P = 0.05). Normobaric oxygen and carbogen did not change tumour oxygenation significantly. Sympathetic blockade with bretylium before HBC exposure improved oxygenation significantly more than HBC alone (low pO2 0.55-0.17, median pO2 4-17 mmHg). HBO and hyperbaric carbogen improved tumour oxygenation in this model, while normobaric oxygen or carbogen had no effect. Sympathetic-mediated vasoconstriction during hyperbaric carbogen caused it to be less effective than HBO. This mechanism also appeared to operate during normobaric carbogen breathing.


British Journal of Cancer (1995) 72, 1120-1124

© 1995 Stockton Press All rights reserved 0007-0920/95 $12.00

### The mechanisms by which hyperbaric oxygen and carbogen improve tumour oxygenation

DM Brizel[1], S Lin[1], JL Johnson[2], J Brooks[1], MW Dewhirst[1] and CA Piantadosi[3]

[1]*Department of Radiation Oncology,* [2]*Division of Biometry, Department of Family Medicine and* [3]*Department of Medicine, Duke University Medical Center, Durham, North Carolina 27710, USA*

**Summary** Hyperbaric oxygen (HBO) has been proposed to reduce tumour hypoxia by increasing the amount of dissolved oxygen in the plasma. That this actually occurs has not been verified experimentally. This study was performed to explore changes in tumour oxygenation induced by treatment with normobaric and hyperbaric oxygen and carbogen. R3230Ac mammary adenocarcinomas were implanted into Fischer 344 rats. Arterial blood gases, blood pressure and heart rate were monitored. Tumour oxygenation was measured polarographically in five sets of animals. They received either normobaric 100% oxygen, hyperbaric (3 atmospheres; atm) 100% oxygen, normobaric carbogen or hyperbaric (3 atm) carbogen (HBC) ± bretylium. HBO reduced the mean level of low $pO_2$ values (<5 mmHg) from 0.49 to 0.07 ($P = 0.0003$) and increased the average median $pO_2$ from 8 mmHg to 55 mmHg ($P = 0.001$). HBC reduced the level of low $pO_2$ values from 0.82 to 0.51 ($P = 0.002$) and increased median $pO_2$ from 2 mmHg to 6 mmHg ($P = 0.05$). Normobaric oxygen and carbogen did not change tumour oxygenation significantly. Sympathetic blockade with bretylium before HBC exposure improved oxygenation significantly more than HBC alone (low $pO_2$ 0.55-0.17, median $pO_2$ 4-17 mmHg). HBO and hyperbaric carbogen improved tumour oxygenation in this model, while normobaric oxygen or carbogen had no effect. Sympathetic-mediated vasoconstriction during hyperbaric carbogen caused it to be less effective than HBO. This mechanism also appeared to operate during normobaric carbogen breathing.

**Keywords**: oxygen; hyperbaric oxygen; carbogen; tumour oxygenation; carbon dioxide; polarographic microelectrode

Thomlinson and Gray (1955) provided indirect evidence that hypoxic, radioresistant cells existed in human tumours in the 1950s. Their work led to the development of the concept of chronic, diffusion-limited hypoxia. Some of the earliest clinical efforts that were designed to overcome chronic hypoxia utilised hyperbaric oxygen (HBO). The rationale for these trials was that higher concentrations of dissolved oxygen in the plasma would provide more gas at the capillary level and allow diffusion to occur farther into otherwise hypoxic tissue regions.

Clinical trials of HBO were initiated in the 1950s and 1960s (Brady *et al.*, 1981; Henk, 1986; Dische, 1991). During this period, several investigators measured tumour oxygenation with simple polarographic microelectrode systems. They showed that HBO improved tumour oxygenation in animals and humans (Jamieson and van den Brenk, 1963, 1965).

The effects of carbogen (95% oxygen/5% carbon dioxide) breathing were also studied. There were two reasons for using carbogen: the carbon dioxide component would help to maintain tumour blood flow by counteracting oxygen-induced vasoconstriction and also increase oxygen delivery by shifting the haemoglobin-oxygen dissociation curve to the right (Rojas, 1991). Some studies showed that carbogen breathing improved tumour oxygenation and blood flow while others did not (Kruuv *et al.*, 1967; Inch *et al.*, 1970). These studies were primarily descriptive in nature and did not explore potential mechanisms that might explain the experimental data.

A major limitation of the early polarographic measurement studies was the inability to sample more than a few points in any given tumour. The development of a computer-controlled polarographic device ($pO_2$ histograph, Eppendorf, Hamburg, Germany) has led to a resurgence in the measurement of tumour oxygenation. Rapid *in situ* measurement of multiple points within a tumour is now possible.

Correspondence: DM Brizel, Department of Radiation Oncology, Box 3085, Duke University Medical Center, Durham, NC 27710, USA
Received 20 December 1994; revised 26 June 1995; accepted 7 July 1995

Recently, some investigators have reintroduced carbogen into the clinic to attempt to improve tumour oxygenation. Falk *et al.* (1992) have demonstrated that carbogen transiently improves tumour $pO_2$ in humans. Whether or not this improvement is greater than that which would result from pure oxygen is not known.

The study reported here was performed in an animal tumour model to measure changes in tumour oxygenation induced by treatment with oxygen and carbogen under normobaric and hyperbaric conditions. Based on the existing data, our hypothesis was that HBC would increase tissue oxygenation to a greater extent than the other modalities. The study was also designed to evaluate possible physiological mechanisms that could explain any observed differences.

### Materials and methods

### *Animal model*

Female Fischer 344 rats weighing 150-170 g had mammary adenocarcinomas (R3230Ac) implanted subcutaneously into the left flank. Tumour size varied from 10-25 mm in diameter. Animals were anaesthetised with intraperitoneal injections of Nembutal (pentobarbital sodium) at a dose of 40 mg $kg^{-1}$ body weight. Tracheostomy was performed to allow mechanical ventilation at a rate of 50-60 breaths per min. An arterial line was placed into the right femoral artery to monitor blood pressure. The animals were placed on a warming pad to maintain body temperature close to 37°C.

### *Tumour oxygen measurement*

Tumour oxygenation was measured with the Eppendorf $pO_2$ histograph. A two-point calibration of the electrode was performed immediately before and after each series of measurements using buffered 0.9% sodium chloride, pH 7.8, which was equilibrated alternately with room air (21% oxygen) and pure nitrogen under normobaric conditions or 7% oxygen and pure nitrogen under hyperbaric conditions at 3 atm.

After the initial calibration, a silver–silver chloride reference electrode was inserted into the right flank of the animal and polarised to a voltage of −700 mV. A small incision was made in the skin over the tumour, and the electrode was inserted approximately 1 mm into the tumour. The electrode was allowed to stabilise. It was then advanced into the tumour in a stepwise fashion consisting of 0.7 mm forward and then 0.3 mm backward. Serial measurements were thus obtained with a net distance of 0.4 mm between points. Measurements were taken along 4–6 tracks at various locations within each tumour until 100 data points were obtained. The electrode was then recalibrated. Arterial blood gas values, heart rate and blood pressure were recorded continuously during each measurement series both under control and experimental conditions. The average values were computed for each parameter.

### *Experimental protocol*

Five sets of experimental manipulations were performed in this study. They consisted of normobaric 100% oxygen, hyperbaric (3 atm) 100% oxygen, normobaric carbogen and hyperbaric (3 atm) carbogen. After preliminary analysis of these experiments a fifth set of animals received an intravenous injection of bretylium followed by exposure to hyperbaric carbogen.

Each animal underwent control tumour $pO_2$ measurements while breathing normobaric room air. The mechanical ventilator was adjusted so that the blood gas values would fall into a 'normal' range (pH approximately 7.45–7.55; $pO_2$ $\geqslant$60 mmHg; and $pCO_2$ approximately 30 mmHg). Each animal therefore served as its own control and no animal received more than one type of experimental treatment.

### *Treatment with normobaric oxygen or carbogen*

Following the control measurements, the ventilator was set to provide 100% oxygen or carbogen for the animal while the breathing rate was not altered. A series of 100 measurements was obtained after the animal had breathed oxygen or carbogen for 5 min. A second set of measurements was obtained after 25 min in the animals that were breathing carbogen.

### *Treatment with hyperbaric oxygen*

The entire apparatus, including the ventilator, the animal and the $pO_2$ histograph, were set up inside a large hyperbaric chamber. The chamber was large enough for the investigators (DB, SL, JB) to accompany the animals during the experiments. Control measurements were performed under normobaric conditions. The chamber was then pressurised to 3 atm (approximately 2280 mmHg). Ambient concentration of oxygen in the chamber was 7% while the animals breathed 100% oxygen from the ventilator. The electrode was recalibrated with 7% oxygen (21% surface equivalent) and pure nitrogen. A series of 100 measurements was made after the animal had breathed pure oxygen under hyperbaric conditions for 5 min. The probe was recalibrated afterwards, and then the chamber was decompressed to 1 atm.

### *Treatment with hyperbaric carbogen*

The procedure was similar to that for the hyperbaric oxygen experiment, but two sets of measurements were made, at 5 min and 25 min after the animal started carbogen breathing.

### *Treatment with bretylium and hyperbaric carbogen*

Animals received an i.v. infusion of bretylium tosylate (Bretylol, Du Pont Pharmaceuticals, Wilmington, DE, USA; 0.4 ml of a 17 mg ml$^{-1}$ solution) over a 20 min period to provide a blockade of the sympathetic nervous system. The hyperbaric chamber was pressurised, and the probe was calibrated. Measurements were performed 20–25 min after completion of the bretylium infusion and approximately 5 min after initiation of carbogen breathing. A second series of measurements was not performed in this group of animals as the required extra time at pressure would have necessitated a prolonged decompression period for the investigators.

### *Statistical considerations*

Paired *t*-tests were used to compare control and experimental measurements within the same group. Repeated measures analysis of variance was used to determine if there were significant differences between the different experimental groups. To adjust for differences in tumour volume between experimental groups, models were fit including tumour volume as a covariate. These models indicated that adjustment was not necessary and tumour volume was not included in the final models. Models were also fit using mean arterial blood pressure, heart rate and arterial $pO_2$ as time-dependent covariates. Adjusting for these variables also had little effect on the results.

### Results

A frequency histogram that illustrates overall patterns of oxygenation for the HBO, HBC and bretylium/HBC groups is shown in Figure 1. Table I summarises the changes in tumour oxygenation that resulted from the different therapeutic manipulations. No significant changes in the percentage of low $pO_2$ values (<5 mmHg) or median $pO_2$ resulted from treatment with NBO or NBC. Highly significant improvement in both of these parameters occurred after 5 min of HBO breathing. The mean low $pO_2$ fraction (LF) decreased from 0.49 to 0.07 while the average median $pO_2$ increased from 8 mmHg to 55 mmHg ($P = 0.0003$ and 0.001 respectively). HBC reduced LF from 0.82 to 0.59 at 5 min. An additional 20 min of HBC breathing led to a further decrease to 0.51 ($P = 0.003$ and 0.002 respectively). Median $pO_2$ was unchanged at 5 min but increased to 6 mmHg at 25 min ($P = 0.05$).

The baseline LFs of the HBO animals were significantly lower than those of the HBC group ($P < 0.001$). Therefore, the changes in LF at 5 min were compared between the two sets of animals. The change of 0.42 for HBO *vs* 0.23 for HBC was of borderline significance ($P = 0.07$). Adjustments for differences in tumour volume, mean arterial pressure, heart rate and arterial $pO_2$ (Table II) did not affect this comparison. Nonetheless, the fact that HBC did not improve tumour oxygenation more than HBO was somewhat unexpected.

**Figure 1** Frequency histograms illustrating the overall distributions of tumour $pO_2$ for the groups of animals receiving hyperbaric oxygen, hyperbaric carbogen (after 5 min of breathing) and bretylium followed by hyperbaric carbogen. The two shaded columns at the left indicate the fraction of low $pO_2$ measurements (<5 mmHg) for each group.

**Table I** Summary of changes in tumour oxygenation

| *Treatment* | n | *Low* $pO_2$ | P-*value* | *Median* $pO_2$ | P-*value* |
|---|---|---|---|---|---|
| Room air | 11 | 0.49 | | 8 | |
| HBO | | 0.07 | 0.0003 | 55 | 0.001 |
| Room air | 8 | 0.53 | | 4 | |
| NBO | | 0.68 | 0.17 | 3 | 0.46 |
| Room air | 8 | 0.71 | | 1 | |
| NBC (5 min) | | 0.53 | 0.12 | 4 | 0.20 |
| NBC (25 min) | | 0.62 | 0.30 | 2 | 0.64 |
| Room air | 8 | 0.82 | | 2 | |
| HBC (5 min) | | 0.59 | 0.003 | 2 | 0.15 |
| HBC (25 min) | | 0.51 | 0.002 | 6 | 0.05 |
| Room air | 4 | 0.55 | | 4 | |
| Bretylium + HBC | | 0.17 | 0.01 | 17 | 0.09 |

HBO, hyperbaric 100% oxygen; NBO, normobaric 100% oxygen; NBC, normobaric carbogen; HBC, hyperbaric carbogen. Low $pO_2$, proportion of measured points with $pO_2 < 5$ mmHg averaged across all animals in individual experimental groups. Median $pO_2$, the median value of individual median $pO_2$ values in each group.

**Figure 2** (**a**) Relative changes in the proportion of low $pO_2$ values (<5 mmHg) for the animals receiving hyperbaric oxygen ( ▨ ), hyperbaric carbogen ( ▧ , after 5 min of breathing) and bretylium followed by hyperbaric carbogen ( ■ ). The baseline for each group has been normalised to 1 ( □ ) to allow a better comparison among the different groups. (**b**) Relative changes in the median tumour $pO_2$ for the animals receiving hyperbaric oxygen ( ▨ ), hyperbaric carbogen ( ▧ , after 5 min of breathing) and bretylium followed by hyperbaric carbogen ( ■ ). The baseline for each group has been normalised to 1 to allow a better comparison among the different groups.

The fact that HBC did not improve tumour oxygenation more than HBO led us to consider that the carbon dioxide may have been eliciting a sympathetic reflex vasoconstriction. Consequently, two additional sets of experiments were performed in which animals received bretylium to assess the effect of sympathetic blockade before carbogen exposure. In these experiments, median $pO_2$ increased from 4 mmHg to 17 mmHg ($P = 0.09$), and LF decreased from 0.55 to 0.17 ($P = 0.02$). The improvements in these oxygenation parameters with the addition of bretylium compared with those which were seen with HBC alone were highly significant ($P = 0.01$ and $P < 0.001$ respectively). The improvements in tumour oxygenation from bretylium/HBC were not significantly different from those seen with HBO ($P = 0.16$). The augmentation in oxygenation that resulted from pretreatment with bretylium was particularly striking in the context of the reduction in mean arterial pressure (MAP) that this drug caused. MAP fell from 112 mmHg to 65 mmHg ($P = 0.001$).

Figure 2a compares the relative changes in LF for the HBO-, HBC- and bretylium/HBC-treated tumours. The baseline LFs were normalised to 1, since this parameter varied among the different groups, in order to more easily compare the changes in oxygenation. Similarly, Figure 2b shows the relative change in median $pO_2$ after 5 min of exposure to HBO, HBC and bretylium/HBC.

### Discussion

Tumour tissue oxygenation is the net outcome of a complex series of processes that influence oxygen supply and demand. Hypoxia results when the supply is inadequate to meet demand. Many efforts have been directed towards overcoming tumour hypoxia because of the relative radioresistance of hypoxic cells. Two approaches have commonly been employed focusing on augmentation of tumour oxygenation or selective targeting of hypoxic regions. Examples of the former include the use of perfluorocarbons, blood transfusions, oxygen and/or carbogen breathing during treatment, and HBO (Brady *et al.*, 1981; Henk, 1986; Lustig *et al.*, 1989, 1990; Dische, 1991; Rockwell *et al.*, 1991). The latter would include the use of nitroimidazoles and bioreductive agents

**Table II** Tumour size and physiological parameters

| *Treatment* | n | *Diameter* | *MAP* | *HR* | pa$O_2$ | pa$CO_2$ |
|---|---|---|---|---|---|---|
| Room air | 11 | 17 ± 3.7 | 128 | 460 | 74 | 28 |
| HBO | | | 129 | 460 | 1455 | 29 |
| Room air | 8 | 15 ± 3.6 | 136 | 463 | 70 | 23 |
| NBO | | | 146 | 505 | 395 | 20 |
| Room air | 8 | 18 ± 2.5 | 134 | 454 | 79 | 23 |
| NBC | | | 133 | 467 | 503 | 43 |
| Room air | 8 | 17 ± 2.6 | 136 | 460 | 76 | 23 |
| HBC | | | 128 | 460 | 1372 | 91 |
| Room air | 4 | 14 ± 1.7 | 112 | 450 | 73 | 28 |
| Bretylium/HBC | | | 65 | 455 | 1365 | 95 |

All data represent median values except diameter which is mean ± s.d. MAP, mean arterial pressure; HR, heart rate; $paO_2$, arterial $pO_2$; $paCO_2$, arterial $pCO_2$.

such as misonidazole, mitomycin C or SR-4233 (Rockwell, 1992).

Secomb *et al.* (1995) recently analysed the effects of oxygen supply and demand on tumour hypoxic fraction, which they defined as the proportion of $pO_2$ measurements <3 mmHg. The model used by these investigators incorporated blood flow rate, blood oxygen content and oxygen consumption rate into the calculation of the hypoxic fraction of a given tumour region. The vascular geometry that they used was taken from morphological observations of the R3230AC tumour, the same type used in the current studies. The other parameters also were based on experimentally derived data. The hypoxic fraction could be eliminated by reduction of the oxygen consumption rate by 30%. On the supply side, however, flow rate had to be increased 4-fold and arterial $pO_2$ ($paO_2$) by a factor of 11 in order to eliminate tumour hypoxia.

Normobaric oxygen and carbogen caused no significant change in tumour oxygenation in the current study while HBO and HBC led to improvement. Both NBO and NBC increased $paO_2$ by a factor of 5-6 (395 mmHg for NBO and 503 mmHg for NBC). HBO and HBC increased it by a factor of approximately 20. If we redefined LF in the current study by Secomb *et al.*'s (1995) 3 mmHg criterion, then breathing normobaric oxygen, normobaric carbogen (measured at 5 min), HBO and bretylium/HBC resulted in fractions of 0.42, 0.44, 0.09 and 0.03 respectively. These changes in $paO_2$ and the corresponding reductions in LF are consistent with what is predicted by the Secomb model.

Ongoing clinical trials are utilising carbogen to attempt to improve tumour oxygenation. The rationale for the use of the 95% oxygen component is to deliver more oxygen to the tumour. The carbon dioxide is supposed to maintain tumour blood flow via peripheral vasodilation and shifting of the haemoglobin-oxygen dissociation curve to the right, which favours oxygen unloading in the tumour (Rojas, 1991). Martin *et al.* (1993) analysed the effect of carbogen breathing on tumour oxygenation with the $pO_2$ histograph in head and neck cancer patients with metastases to cervical lymph nodes. Median $pO_2$ improved in most tumours. However, the backward movement of the probe (0.1 mm) and the net forward probe movement (0.2 mm) were less than that used in the current study (0.3 mm and 0.4 mm respectively) and are less than that recommended by the manufacturer (M Gunderoth, personal communication). The shorter net distance between measured points increases the chance that haemorrhage from a previous measurement could contaminate subsequent measurements. In such a situation, the higher $paO_2$ from any inspired gas could erroneously lead one to conclude that tissue $pO_2$ was increased.

Siemann *et al.* (1977) showed in an animal model that a preirradiation carbogen breathing time (PIBT) of 10 min produced a maximum radiosensitisation. As PIBTs increased up to 90 min, radiosensitivity returned to the baseline level seen with air breathing. Similarly, Inch *et al.* (1970) showed that a 0.5 min carbogen PIBT more effectively radiosensitised than a 12 min PIBT. Falk *et al.* (1992) recently observed the same general sequence of changes in a clinical trial where human patients underwent serial sets of $pO_2$ measurements with the $pO_2$ histograph while breathing carbogen. Median tumour $pO_2$ increased in 12 out of 17 patients after 8-12 min of carbogen breathing. With additional carbogen exposure, tumour oxygenation returned towards baseline in many of these patients. Similarly, in the current study, LF decreased and median $pO_2$ increased, albeit in a statistically non-significant fashion, during the first 5 min of normobaric carbogen exposure and then returned towards the baseline control values with additional carbogen breathing. These time-dependent findings are similar to those of the previously cited investigators (Jamieson and van den Brenk, 1965; Inch *et al.*, 1970; Siemann, 1977; Falk *et al.*, 1992). They differ from a majority of other investigators' findings of carbogen-mediated increases in tumour oxygenation and/or radiosensitisation (Kruuv *et al.*, 1967; Rockwell *et al.*, 1991; Rojas, 1991; Grau *et al.*, 1992; Chaplin *et al.*, 1993; Martin *et al.*, 1993; Horsman *et al.*, 1994; Siemann *et al.*, 1994). Hyperbaric carbogen did improve tumour oxygenation significantly, but, contrary to our expectations, the magnitude of improvement did not exceed that caused by HBO.

The effects of normobaric and hyperbaric carbogen on tumour oxygenation that we observed do not fit into Secomb *et al.*'s (1995) model even though the latter increased $paO_2$ from 76 mmhg to 1372 mmHg. The effect of these two gases can be interpreted, however, by consideration of the direct and indirect effects of carbon dioxide exposure on the vasculature. NBC and HBC caused $paCO_2$ to increase to 43 mmHg and 91 mmHg respectively. Carbon dioxide inhalation provides a very potent stimulus in the brainstem that results in activation of the sympathetic nervous system (Richardson *et al.*, 1961). Blood flow to the forearms decreases in patients breathing high concentrations of carbon dioxide (Blair *et al.*, 1961). Blood flow increases, however, if a nerve block is performed beforehand (Hampson *et al.*, 1987). Conversely, carbon dioxide causes vasodilation and decreased total peripheral resistance (TPR) in sympathectomised limbs but vasoconstriction and increased TPR in non-sympathectomised limbs (Steck and Gellhorn, 1939). Thus, the direct effect of carbon dioxide breathing is peripheral vasodilation and increased blood flow, but this is masked by indirect, sympathetic effects that override the direct effects of carbon dioxide and result in vasoconstriction and a net decrease in flow.

That HBC was not more effective than HBO in improving tumour oxygenation would appear to be the result of adrenergic stimulation from the inspired carbon dioxide. HBC breathing preceded by the injection of bretylium, which is a potent sympatholytic agent, was significantly superior to HBC alone. This fact provides strong, direct evidence to support the argument that central sympathetic control mechanisms interfere with the direct dilator effects of carbon dioxide. The pronounced decrease in MAP caused by bretylium indicates that the dose of this agent that we used provided a complete sympathetic blockade. The improvement in tumour oxygenation with bretylium/HBC is even more striking in view of the extent of this decrease in MAP.

The direct vasodilatory effects and the indirect sympathetic vasoconstrictor effects of carbon dioxide also offer a possible explanation for the normobaric carbogen-induced changes observed both in preclinical studies, including ours, and in human investigations (Jamieson and van den Brenk, 1963, 1965; Kruuv *et al.*, 1967; Inch *et al.*, 1970; Siemann *et al.*, 1977; Falk *et al.*, 1992). An initial improvement in tumour oxygenation followed by a return towards baseline could occur if the direct effects predominated initially and were superceded by the indirect effects with additional carbogen breathing.

Many investigators have studied tumour oxygenation in awake patients or animals while the animals in the current study were anaesthetised. The anaesthesia could have adversely affected blood flow and oxygen delivery. This is not likely, however, as the baseline haemodynamic parameters (MAP, HR, arterial $pO_2$) for these animals were not different from what has been reported when they are awake (Smith *et al.*, 1985). Nembutal can also depress respiratory drive which could adversely affect tissue oxygenation. We also think that this is unlikely to have been a problem because the animals in this study were mechanically ventilated at rates at which they breathe when conscious. Furthermore, the experimental gases caused no major changes in any of the haemodynamic parameters except in the case of bretylium/HBC where the intent was sympathetic blockade and where a decrease in MAP was expected. We believe that performing oxygen measurements on anaesthetised animals is as valid as doing them on restrained, awake animals where stress and discomfort could cause catecholamine-induced changes in blood flow and oxygenation.

Baseline tumour oxygenation was best in the HBO animals and became progressively worse as different animals were exposed to NBO, NBC and HBC. The greater reduction in LF in the HBO animals compared with the HBC animals was of borderline significance. It is possible, however, that improving tumour oxygenation is more difficult in very hypoxic tumours.

The tumours in the NBO, NBC and HBC groups may also have been more necrotic than the HBO tumours even though there were no differences in tumour diameter. Polarographic electrodes cannot distinguish between hypoxic, viable tissue and necrotic tissue. Under conditions of necrosis, no therapeutic manipulation would be expected to improve oxygenation.

The number of measurement tracks performed does represent a potentially confounding variable in the interpretation of the data. Since 4–6 tracks were obtained per procedure and since each animal served as its own control, from 8–18 tracks (2–3 determinations × 4–6 tracks) could have been made in any given tumour. Oedema and haemorrhage from the trauma could have led to an overestimation or underestimation of tumour oxygenation. It is likely, however, that this type of problem would have affected all of the different experimental groups equally.

### Conclusion

The present study demonstrated that hyperbaric oxygen and hyperbaric carbogen improved tumour oxygenation in the R3230AC mammary carcinoma implanted in the hindlimb. Sympathetic-mediated vasoconstriction during carbogen breathing prevented the latter from being more effective than HBO. This mechanism also appeared to operate during normobaric carbogen breathing. It may also explain the findings obtained in the human studies of carbogen breathing. We will evaluate the extent to which these manipulations of tumour oxygenation influence the response to ionising irradiation in future animal studies.

**Acknowledgements**

This work was supported by grants: RO1 CA40355 and PO1 HL-42444-05.

### References

BLAIR DA, GLOVER WE AND RODDIE IC. (1961). Vasomotor responses in the human arm during leg exercise. *Circ. Res.*, **9**, 264–274.

BRADY LW, PLENK HP, HANLEY JA, GLASSBURN JR, KRAMER S AND PARKER RG. (1981). Hyperbaric oxygen therapy for carcinoma of the cervix – stages IIB, IIIA, IIIB, and IVA: results of a randomized study by the Radiation Therapy Oncology Group. *Int. J. Radiat. Oncol. Biol. Phys.*, **7**, 991–998.

CHAPLIN DJ, HORSMAN MR AND SIEMANN DW. (1993). Further evaluation of nicotinamide and carbogen as a strategy to reoxygenate hypoxic cells *in vivo*: importance of nicotinamide dose and pre-irradiation breathing time. *Br. J. Cancer*, **68**, 269–273.

DISCHE S. (1991). What have we learnt from hyperbaric oxygen? *Radiother. Oncol.*, **20** (suppl.), 71–74.

FALK SJ, WARD R AND BLEEHEN NM. (1992). The influence of carbogen breathing on tumor tissue oxygenation in man evaluated by computerized $pO_2$ histography. *Br. J. Cancer*, **66**, 919–924.

GRAU C, HORSMAN MR AND OVERGAARD J. (1992). Improving the radiation response in a C3H mouse mammary carcinoma by normobaric oxygen or carbogen breathing. *Int. J. Radiat. Oncol. Biol. Phys.*, **22**, 415–419.

HAMPSON NB, JOBSIS-VANDERVLIET FF AND PIANTADOSI CA. (1987). Skeletal muscle oxygen availability during respiratory acid–base disturbances in cats. *Respir. Physiol.*, **70**, 143–158.

HENK JM. (1986). Late results of a trial of hyperbaric oxygen and radiotherapy in head and neck cancer: a rationale for hypoxic cell sensitizers? *Int. J. Radiat. Oncol. Biol. Phys.*, **19**, 97–102.

HORSMAN MR, NORDSMARK M, KHALIL AA, HILL SA, CHAPLIN DJ, SIEMANN DW AND OVERGAARD J. (1994). Reducing acute and chronic hypoxia in tumors by combining nicotinamide with carbogen breathing. *Acta Oncol.*, **33**(4): 371–376.

INCH WR, MCCREDIE JA AND SOUTHERLAND RM. (1970). Effect of duration of breathing 95% oxygen plus 5% carbon dioxide before X-irradiation on cure of C3H mammary tumor. *Cancer*, **4**, 926–931.

JAMIESON D AND VAN DEN BRENK HAS. (1963). Comparison of oxygen tensions in normal tissues and Yoshida sarcoma of the rat breathing air or oxygen at 4 atmospheres. *Br. J. Cancer*, **17**, 70–78.

JAMIESON D AND VAN DEN BRENK HAS. (1965). Oxygen tension in human malignant disease under hyperbaric conditions. *Br. J. Cancer*, **19**, 139–150.

KRUUV J, INCH WR AND MCCREDIE JA. (1967). Effects of breathing gases containing oxygen and carbon dioxide at 1 and 3 atmospheres pressure on blood flow and oxygenation of tumors. *Can. J. Physiol. Pharmacol.*, **45**, 49–56.

LUSTIG R, LOWE N, ROSE C, HAAS J, KRASNOW S, SPAULDING M AND PROSNITZ L. (1989). Phase I/II study fluosol and 100% oxygen as an adjuvant to radiation in the treatment of locally advanced non-small cell carcinoma of the head and neck. *Int. J. Radiat. Oncol. Biol. Phys.*, **16**, 1587–1593.

LUSTIG R, LOWE N, PROSNITZ L, SPAULDING M, COHEN M, STITT J AND BRANNON R. (1990). Fluosol and oxygen breathing as an adjuvant to radiation therapy in the treatment of locally advanced non-small cell carcinoma of the lung: results of a phase I/II study. *Int. J. Radiat. Oncol. Biol. Phys.*, **19**, 97–102.

MARTIN L, LARTIGAU E, WEEGER P, LAMBIN P, LE RIDANT AM, LUSINCHI A, WIBAULT P, ESCHWEGE F, LUBOINSKI B AND GUICHARD M. (1993). Changes in the oxygenation of head and neck tumors during carbogen breathing. *Radiother. Oncol.*, **27**, 123–130.

RICHARDSON DW, WASSERMAN AJ AND PATTERSON JL. (1961). General and regional circulatory responses to change in blood pH and carbon dioxide tension. *J. Clin. Invest.*, **40**, 31–43.

ROCKWELL S. (1992). Use of hypoxia-directed drugs in the therapy of solid tumors. *Semin. Oncol.*, **19** (suppl. 11), 29–40.

ROCKWELL S, KELLEY M, IRVIN CG, HUGHES CS, PORTER E, YABUKI H AND FISCHER JJ. (1991). Modulation of tumor oxygenation and radiosensitivity by a perfluorooctylbromide emulsion. *Radiother. Oncol.*, **22**(2), 92–98.

ROJAS A. (1991). Radiosensitization with normobaric oxygen and carbogen. *Radiother. Oncol.*, **20** (suppl.), 65–70.

SECOMB TW, HSU R, ONG ET, GROSS JF AND DEWHIRST MW. (1995). Analysis of the effects of oxygen supply and demand on hypoxic fraction in tumors. *Acta Oncol.* **34**(3), 313–316.

SIEMANN DW, HILL RP AND BUSH RS. (1977). The importance of the pre-irradiation breathing times of $O_2$ and carbogen (95% $O_2$/5% $CO_2$) on the *in vivo* radiation response of a murine sarcoma. *Int. J. Radiat. Oncol. Biol. Phys.*, **5**, 1963–1970.

SIEMANN DW, HORSMAN MR AND CHAPLIN DJ. (1994). The radiation response of KHT sarcomas following nicotinamide treatment and carbogen breathing. *Radiother. Oncol.*, **31**, 117–122.

SMITH TL, OSBORNE SW AND HUTCHINS PM. (1985). Longterm micro- and macrocirculatory measurements in conscious rats. *Microvasc. Res.*, **29**, 360–370.

STECK IE AND GELLHORN E. (1939). The effect of carbon dioxide inhalation on the peripheral blood flow in the normal and in the sympathectomized patient. *Am. Heart J.*, **18**, 206–212.

THOMLINSON RH AND GRAY LH. (1955). The histologic structure of some human lung cancers and the possible implications of radiotherapy. *Br. J. Cancer*, **9**, 537–549.